# Qiliqiangxin capsule attenuates platelet activation and thrombosis by suppressing Ca^2+^ influx and PKC signaling

**DOI:** 10.1186/s12959-025-00823-8

**Published:** 2026-01-02

**Authors:** Liping Han, Wei Zhang, Changyu Huo, Anqi Zhou, Ziru Huang, Yanqi Wang, Biwei Yang, Lihua Cao, Si Zhang, Jiayu Zheng, Rong Xia

**Affiliations:** 1 Department of Transfusion Medicine, Huashan Hospital, Fudan University, Shanghai, 200040 China; 2 Department of Hematology, Zhongshan Hospital Qingpu Branch, Fudan University, Shanghai, 200032 China; 3 NHC Key Laboratory of Glycoconjugate Research, Department of Biochemistry and Molecular Biology, School of Basic Medical Sciences, Fudan University, Shanghai, 200032 China; 4 Department of Cardiac Surgery, Zhongshan Hospital, Fudan University, 180 Fenglin Road, Shanghai, 200032 China; 5 Liver Cancer Institute, Zhongshan Hospital, Fudan University, 180 Fenglin Road, Shanghai, 200032 China; 6 Liver Cancer Institute, Qidong People‘s Hospital Affiliated to Nantong University, Nantong, 226200 China

**Keywords:** Platelet activation, Qiliqiangxin capsules, Thrombosis

## Abstract

**Background:**

Arterial thrombotic diseases are leading causes of global morbidity and mortality, primarily driven by platelet hyperactivity. Qiliqiangxin capsules (QLQX), a traditional Chinese medicine approved in China for the treatment of heart failure, have exhibited potential benefits in reducing cardiovascular death. However, its direct effects on platelet activation and thrombosis remain unclear.

**Methods and results:**

Ex vivo platelet function assays demonstrated that oral QLQX inhibited agonist‑induced aggregation in platelet‑rich plasma (PRP) from chronic heart failure (CHF) patients and dose-dependently suppressed washed platelet aggregation in wild‑type mice. In parallel, QLQX administration attenuated platelet spreading and clot retraction in washed platelets from both patients with CHF and wild-type mice, and reduced ex vivo thrombus formation area in a microfluidic whole‑blood perfusion under arterial shear stress. Flow cytometry analysis revealed that QLQX did not affect the surface expression levels of the major platelet receptors, but reduced the activation of αIIbβ3 and P-selectin exposure induced by thrombin in mouse platelets. In vivo experiments demonstrated that QLQX treatment inhibited FeCl₃-induced thrombus formation in mesenteric arterioles, reduced collagen/epinephrine-induced pulmonary embolism, and mitigated microvascular thrombosis during myocardial ischemia-reperfusion (I/R) injury, without increasing bleeding in mice. RNA sequencing of platelets from QLQX- versus vehicle-treated mice identified differentially expressed genes enriched in the calcium signaling pathway, and functional assays demonstrated that QLQX inhibited agonist-induced platelet Ca²⁺ influx and PKC phosphorylation.

**Conclusion:**

QLQX inhibits platelet activation and thrombosis by targeting Ca²⁺ influx and PKC signaling, supporting its potential therapeutic value in preventing thrombotic complications.

**Supplementary Information:**

The online version contains supplementary material available at 10.1186/s12959-025-00823-8.

## Introduction

Arterial thrombotic diseases, including myocardial infarction and ischemic stroke, remain major causes of morbidity and mortality worldwide [1]. Among the various pathological processes, platelet hyperreactivity and subsequent arterial thrombosis serve as the central drivers [2]. Despite the proven benefits of current antiplatelet therapy in coronary artery disease, residual event and mortality rates remain high, and treatment is often hampered by intolerance and bleeding complications [3–5]. These limitations highlight an unmet need for novel antiplatelet strategies that can optimize antithrombotic efficacy while reducing adverse effects.

Qiliqiangxin capsule (QLQX), a traditional Chinese medicine (TCM) formulation approved by the China Food and Drug Administration for the treatment of heart failure (HF) in 2004, has been widely used in China [6]. Previous studies have demonstrated that QLQX can reduce cardiomyocyte apoptosis, improve cardiac function, restrain myocardial remodeling, and attenuate myocardial fibrosis [7–11]. Multicenter, randomized, placebo-controlled trials have supported its efficacy and safety in patients with CHF [6, 12]. Notably, in a large randomized trial with a median follow-up of 18 months, QLQX treatment reduced major adverse cardiovascular events (MACE) by 22% compared with placebo [12]. Although QLQX has shown cardioprotective effects in CHF, existing research has predominantly focused on its direct effects on cardiomyocytes. Whether QLQX modulates platelet activation and thrombosis remains unclear.

In this study, we demonstrate that QLQX significantly inhibits platelet activation, attenuates thrombosis, and limits myocardial injury as well as microthrombus formation after myocardial ischemia–reperfusion (I/R) injury, without increasing bleeding risk in mice. Mechanistically, QLQX suppresses platelet activation by inhibiting Ca^2+^ influx and protein kinase C (PKC) signaling pathway. These findings collectively suggest that QLQX may be a therapeutic strategy for managing thrombosis.

## Materials and methods

### Human studies

Patients with CHF (18–75 years) were recruited. Inclusion criteria [6, 12]: (1) diagnosis of heart failure for at least 3 months according to the Chinese heart failure diagnosis and treatment guidelines; (2) NYHA cardiac functional grading of II to III, left ventricular ejection fraction (LVEF) ≤ 40%, and serum NT-proBNP level ≥ 450 pg/mL; (3) received standardized medical treatment with a fixed dosage before enrollment, and maintained the same regimen during the study; (4) no use of other TCM herbs. Exclusion criteria: (1) CHF caused by valvular disease, congenital heart disease, pericardial disease, cardiac arrhythmia, or other noncardiogenic factors; (2) cardiogenic shock, malignant tumor, hematopoietic diseases, neuroendocrine system disease, liver dysfunction, or renal dysfunction; (3) pregnancy; (4) QLQX allergy; (5) non-compliance.

Blood samples from enrolled patients with CHF were collected before and 4 weeks after QLQX administration (0.3 g per capsule, four capsules three times daily).

### Animal studies

C57BL/6J mice were obtained from Cyagen Biosciences (Suzhou, China). Animals were maintained under specific pathogen-free conditions in a temperature-controlled facility (22 ± 2 °C), with a 12-hour light/dark cycle at the Department of Laboratory Animal Science, Fudan University. Anesthesia was induced with pentobarbital sodium (50 mg/kg body weight, i.p.) before all invasive procedures unless otherwise specified. All experiments used 6 to 8-week-old mice of both sexes (half-and-half, or 3 males and 2 females for *n* = 5).

All mice were pretreated with QLQX or saline by gavage once daily for 7 consecutive days before experimental procedures. Based on the adult clinical regimen of QLQX (total 3.6 g/day), and using a 70‑kg human reference with body surface area scaling (human‑to‑mouse conversion factor 9.1), the mouse-equivalent dose was calculated as 0.468 g/kg/day [8].

### Reagents

The detailed reagents are listed in Table S1.

### Platelet isolation

Human blood samples were collected from the antecubital vein into tubes containing sodium citrate. Platelet-rich plasma (PRP) was obtained by centrifugation as previously described [13]. Washed platelets were separated from PRP by centrifugation at 700 × g, and the pellet was resuspended in Ca^2+^-free Tyrode’s buffer (138 mM NaCl, 2.7 mM KCl, 2 mM MgCl_2_, 0.42 mM NaH_2_PO_4_, 5 mM glucose, 10 mM HEPES, pH 7.4) containing 0.02 U/mL apyrase.

Following anesthesia with pentobarbital sodium (50 mg/kg body weight, i.p.), mouse blood was collected from the abdominal aorta into syringes containing 3.8% sodium citrate (blood: sodium citrate, 9:1, v/v). PRP was prepared by centrifugation at 270 × g, then at 420 × g to pellet platelets, followed by gentle resuspension in Tyrode’s buffer.

### Routine blood test

Blood samples were obtained via retro-orbital bleeding from mice under anesthesia and collected into EDTA-coated tubes. Subsequently, a complete blood count (CBC) was performed using an automated hematology analyzer (Mindray, China) to assess hematological parameters, including white blood cell count, red blood cell count, platelet count, and mean platelet volume.

### Platelet aggregation

Platelet aggregation in response to agonists was measured using the aggregometers (560CA, Chrono-Log, USA) under stirring conditions (1200 rpm) at 37 °C as described previously [13].

### Platelet spreading

As previously described [13], washed platelets (3 × 10^7^/mL) were seeded onto fibrinogen (100 µg/mL), incubated at 37 °C for the indicated time points, stained with Alexa Fluor 488-phalloidin, imaged by fluorescence microscopy (Olympus BX53, Japan), and quantified using ImageJ (NIH, USA).

### Clot retraction

Clot retraction was initiated by thrombin (1.0 U/mL) at 37 °C, and photographs were taken at the indicated time points. The images were quantified using ImageJ as described previously [14].

### Flow cytometry

For detecting surface expression levels of platelet receptors, including αIIb, β3, GPVI, and GPIbα, washed mouse platelets (3 × 10^7^/mL) were stained with APC-conjugated CD41 antibody, PE-conjugated CD61 antibody, PE-conjugated GPVI antibody, and Alexa Fluor 555-conjugated CD42b antibody, respectively.

To detect P-selectin exposure and the active form of integrin αIIbβ3, washed mouse platelets (3 × 10⁷/mL) were stimulated by thrombin (0.05 U/mL) for 5 min, and then were incubated with PE-conjugated CD62P antibody and PE-conjugated JON/A antibody, respectively.

The samples were analyzed using a flow cytometer (FACSCalibur, BD Biosciences). Data analysis was performed using FlowJo v10.10.0 (BD Biosciences, USA).

### Transmission electron microscopy

Platelet ultrastructure was analyzed by transmission electron microscopy (TEM) according to an established protocol [13]. Briefly, washed platelets were fixed in 2.5% glutaraldehyde prepared in 50 mM cacodylate buffer (pH 7.2) for 1 h at room temperature. Samples were then post-fixed in osmium tetroxide, dehydrated through a graded ethanol series, and embedded in epoxy resin. Ultrathin sections were stained with uranyl acetate and lead citrate and examined using a transmission electron microscope (FEI Tecnai G2 Spirit, USA). Images were acquired digitally, and the platelet area was quantified using ImageJ software (NIH, USA).

### Thrombus formation under flow conditions

Whole blood labeled with FITC-conjugated CD41 antibody was perfused through collagen (100 µg/mL)-coated bioflux plates at a shear stress of 40 dynes/cm² for 5 min) as previously described [14]. Thrombus formation was recorded using a fluorescence microscope and quantified with Bioflux software.

### FeCl_3_-induced thrombosis in mouse mesenteric arteriole

As described previously [14], calcein AM-labeled platelets were injected via the tail vein. After anesthesia, mesenteric arterioles were injured with 10% FeCl_3_ filter paper for 2 min. Thrombus formation was monitored by intravital fluorescence microscopy. The time to first thrombus (> 20 μm) and the occlusion time were recorded.

### Pulmonary embolism model

Under anesthesia, pulmonary embolism was induced via tail vein injection of a collagen (300 µg/kg)/epinephrine (60 µg/kg) mixture [15]. Thirty minutes later, lung tissue was harvested for histological examination. Hematoxylin and eosin (H&E) and anti‑CD42b (platelet marker) staining were performed by Servicebio Technology (Wuhan, China).

### Myocardial I/R model

Mice were anesthetized with 1.5% isoflurane and mechanically ventilated as previously described [13]. A left thoracotomy was performed, and 6 − 0 silk ligated around the left anterior descending (LAD) coronary artery to induce ischemia for 45 min, and then the ligature was removed.

At 48 h post-I/R, the LAD was re-occluded, and 1% Evans blue was injected into the left ventricle. Hearts were excised, rinsed, frozen at -20 °C, sectioned into 1‑mm slices, incubated in 1% TTC at 37 °C, and fixed in 4% paraformaldehyde. Area/area-at-risk (AAR) and infarct size were quantified using ImageJ. For cardiac microthrombi, hearts were fixed and stained with a CD42b antibody. The CD42b‑positive area was quantified using ImageJ.

### Tail bleeding assay

Mice were anesthetized, and the tail was transected 5 mm from the tip and immersed in 10 mL saline at 37 °C. Bleeding time was recorded from transection until bleeding ceased ≥ 2 min, and blood loss was calculated using a standard curve produced from known volumes of mouse blood [16].

### Plasma coagulation tests

Blood was collected from the abdominal aorta under anesthesia and anticoagulated with 3.8% sodium citrate. After centrifugation at 3000 rpm for 15 min to obtain platelet-poor plasma, prothrombin time (PT) and activated partial thromboplastin time (APTT) were determined on an automatic coagulation analyzer (RAC-1830, Rayto, China) according to the manufacturer’s instructions.

### Determination of serum AST, ALT, UA, and BUN

Blood was collected from anesthetized mice via retro-orbital bleeding. After clotting at room temperature for 30 min, the samples were centrifuged at 3000 × g for 15 min to isolate serum. The levels of alanine aminotransferase (ALT), aspartate aminotransferase (AST), uric acid (UA), and blood urea nitrogen (BUN) were measured using respective commercial assay kits, with absorbance read on a Spar Multimode Microplate Reader (Tecan).

### Platelet mRNA sequencing

RNA was extracted from mouse platelets using TRIzol. RNA quality was assessed before transcriptome sequencing (LC-Bio, China). Differentially expressed genes (FDR < 0.05, |fold change| ≥ 2) were identified by DESeq2, and pathways were analyzed via Gene Set Enrichment Analysis (GSEA).

### Calcium mobilization

Washed platelets were loaded with Fura‑2 AM (5 µM) at 37 °C for 30 min and stimulated with agonists at 37 °C for 5 minutes [17]. Fluorescence was recorded using a Duetta fluorescence spectrophotometer (HORIBA Scientific, Japan).

### Western blotting

Platelets were lysed in RIPA buffer supplemented with protease/phosphatase inhibitors, mixed with loading buffer, denatured, resolved by SDS–PAGE, and transferred to PVDF membranes. Membranes were incubated with primary antibodies and HRP‑conjugated secondary antibodies, and signals were captured on Tanon 5200 imaging system.

### Statistical analysis

Data are expressed as mean ± SEM or median (lower to upper quartile) and analyzed with GraphPad Prism 9.0 (GraphPad Software, USA). For two-group comparisons, paired or unpaired Student’s t-tests were used for normally distributed data, and the Mann–Whitney U test for non-normal data. For comparisons among more than two groups, two-way ANOVA was applied, followed by Sidak’s multiple comparisons. *P* < 0.05 was considered statistically significant.

## Results

### QLQX treatment inhibits platelet activation in patients with CHF

**Fig. 1:**
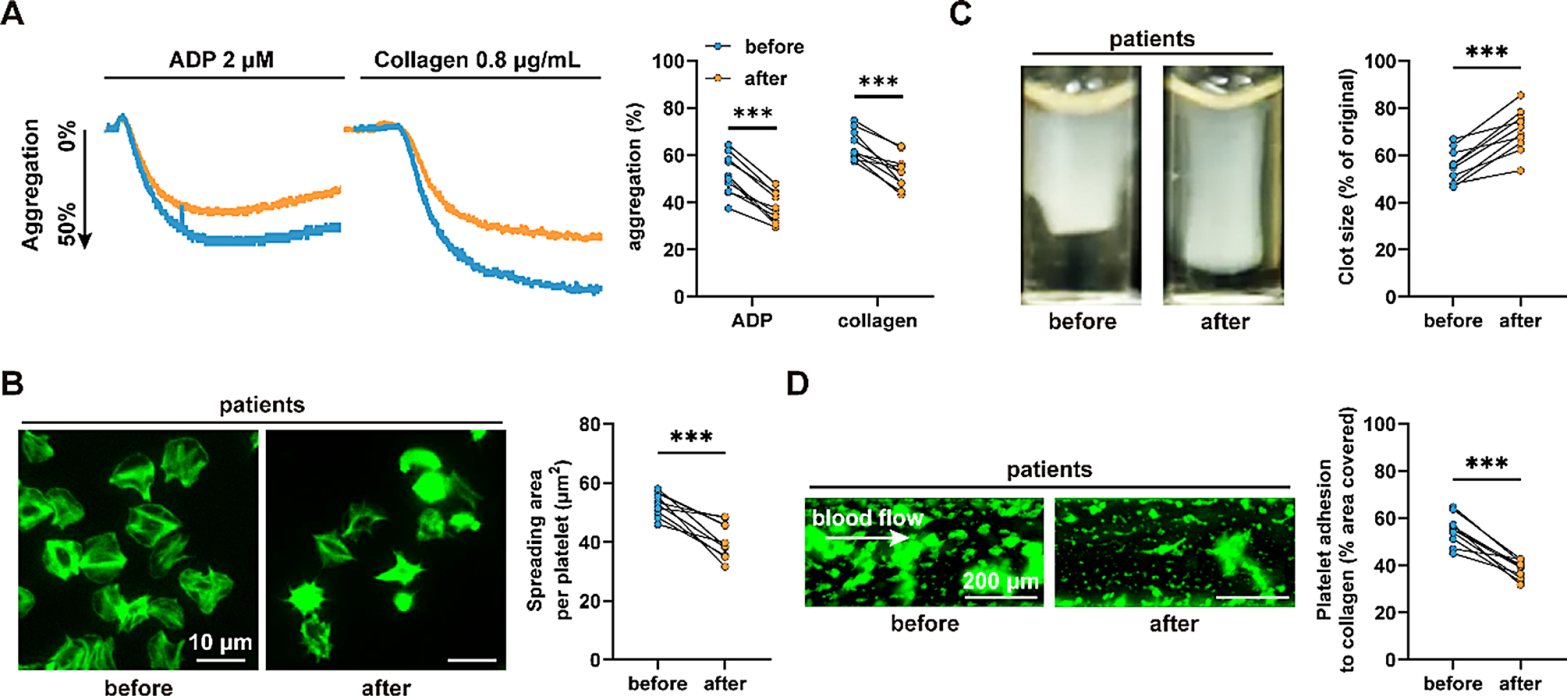
QLQX treatment inhibits platelet activation in patients with CHF. **(A)** 4-week QLQX administration inhibited platelet aggregation in response to ADP (2 µM) and collagen (0.8 µg/mL) in PRP from patients with CHF. **(B-C)** 4-week QLQX administration suppressed platelet spreading on immobilized fibrinogen (100 µg/mL) **(B)** and clot retraction induced by thrombin (1.0 U/mL) **(C)** in patients with CHF. Scale bar = 10 μm. **(D)** Whole blood from patients with CHF exhibited reduced platelet adhesion and ex vivo thrombus formation on immobilized collagen (100 µg/mL) at a shear rate of 1000 s^− 1^ after 4-week QLQX administration. Scale bar = 200 μm. Statistical analyses were performed using a paired two-tailed Student’s t-test. *n* = 10. ***P < 0.001

To investigate the impact of QLQX on human platelet activation, we enrolled 10 patients with CHF who had not previously received QLQX, according to the inclusion criteria. Baseline characteristics are summarized in Table S2. Subjects were instructed to take QLQX (3.6 g/day [12]) for four weeks (duration of a course of treatment) without any other medication status changed. Blood samples were collected before and after QLQX treatment. We found that QLQX inhibited platelet aggregation induced by ADP (2 µM) and collagen (0.8 µg/mL) in patients with CHF (Fig. 1A). Upon activation, integrin αIIbβ3 undergoes conformational changes that initiate an outside-in signaling cascade—a critical process driving platelet spreading and clot retraction, thereby promoting stable thrombus formation [18]. Consistently, oral QLQX attenuated washed platelet spreading on immobilized fibrinogen (Fig. 1B) and clot retraction induced by thrombin (Fig. 1C) in patients with CHF.

Subsequently, we assessed the effect of QLQX administration on ex vivo thrombus formation under arterial shear stress (1000 s⁻¹) using a microfluidic whole-blood perfusion assay. QLQX reduced the thrombus area during the perfusion period in patients with CHF (Fig. 1D).

Collectively, these results demonstrate that QLQX inhibits platelet activation and ex vivo thrombus formation in patients with CHF.

### Administration of QLQX does not exhibit obvious pharmacological toxicity

**Fig. 2:**
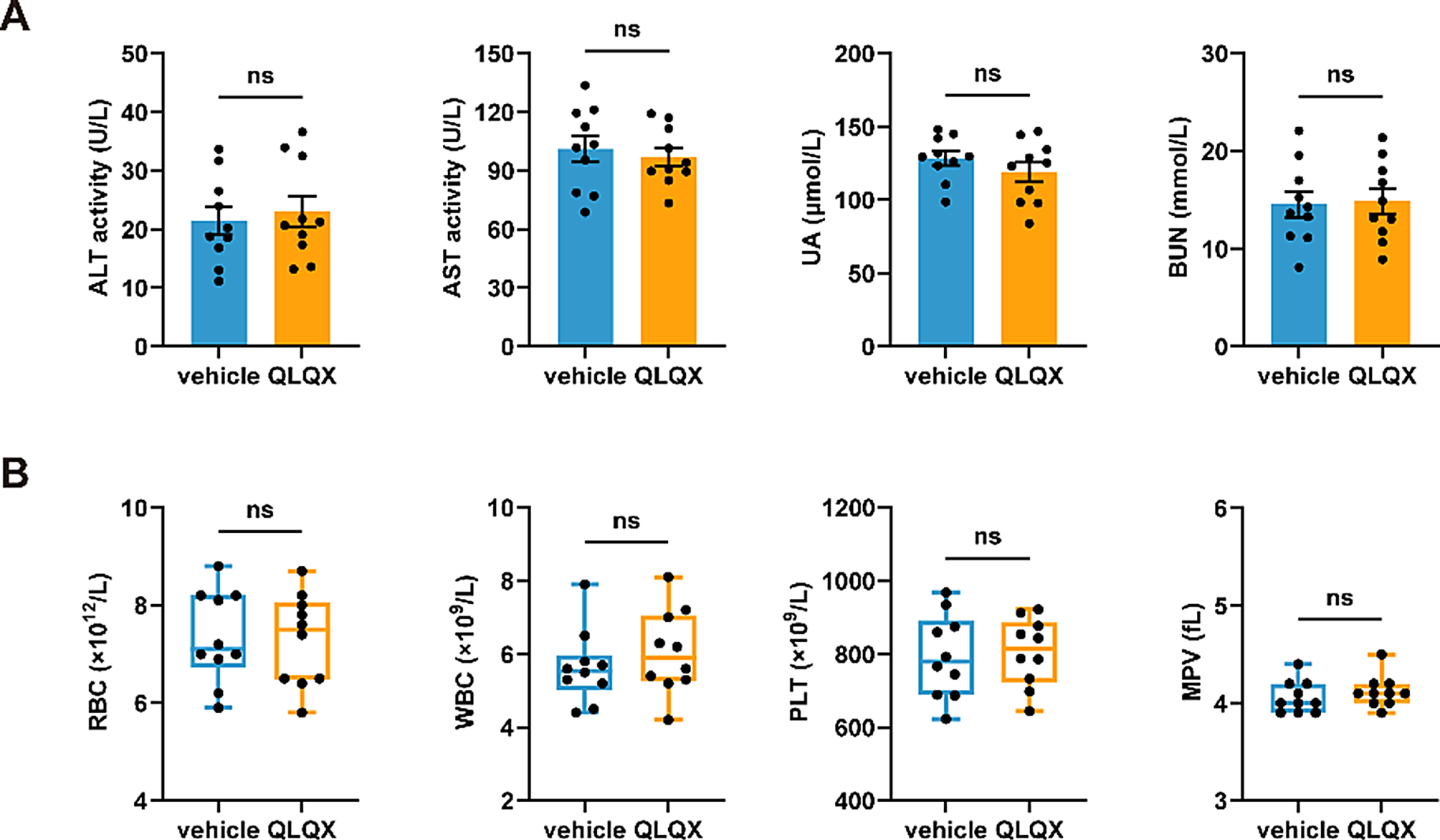
QLQX shows no significant pharmacological toxicity. **(A)** Administration of QLQX to mice at a dose of 0.468 g/kg/day (o.g.) for 7 days did not induce obvious liver or kidney damage. This was determined by analyzing serum biomarkers: alanine aminotransferase (ALT) and aspartate transaminase (AST) for hepatic injury, and uric acid (UA) and blood urea nitrogen (BUN) for renal injury. **(B)** Administering QLQX to mice at a dose of 0.468 g/kg/day for seven days did not affect blood cell count and platelet parameters. RBC, red blood cells; WBC, white blood cells; PLT, platelets; MPV, mean platelet volume. Statistical analyses were performed using an unpaired two-tailed Student’s t test in **(A)** and Mann-Whitney U test in **(B)**. *n* = 10. ns, not significant

To assess the toxicity of QLQX (at the mouse-equivalent dose of the adult clinical regimen, 0.468 g/kg/day) in wild-type mice, whole blood samples were collected after seven days of oral gavage; saline served as the vehicle control. Treatment with QLQX did not cause obvious liver or kidney damage, evidenced by no significant differences in serum levels of alanine aminotransferase (ALT), aspartate aminotransferase (AST), uric acid (UA), and blood urea nitrogen (BUN) between QLQX-treated mice and control mice (Fig. 2A). Automated complete blood count (CBC) analysis revealed comparable white blood cell count, red blood cell count, platelet count, and mean platelet volume (MPV) between QLQX-treated and control mice (Fig. 2B).

### QLQX administration inhibits mouse platelet activation

**Fig. 3:**
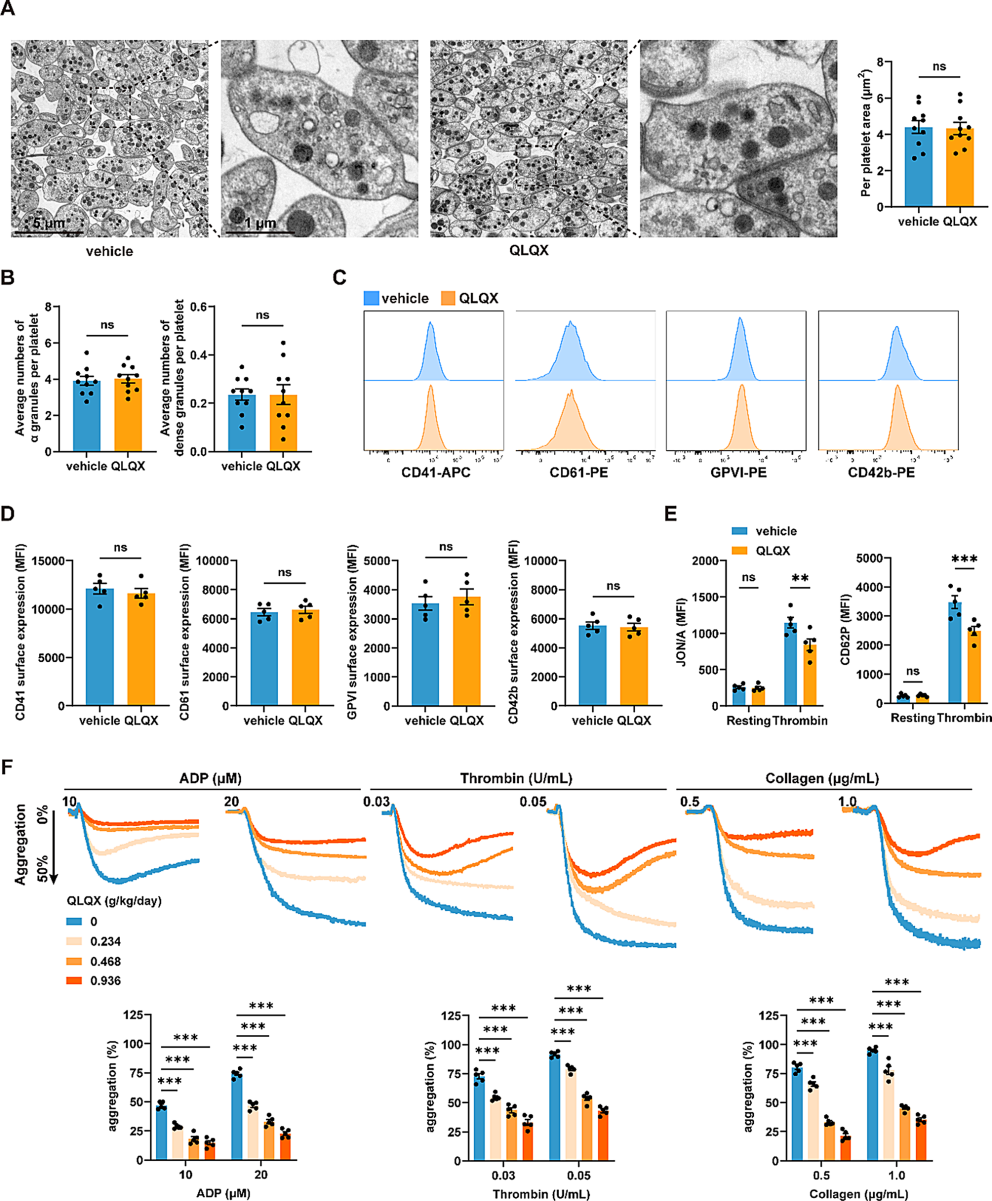
QLQX inhibits platelet activation in mice. **(A-B)** QLQX treatment did not affect platelet ultrastructure in mice. Representative transmission electron microscopy (TEM) images of platelets (**A**, left; Scale bar = 5 μm–1 μm) and quantitative analysis of platelet area (**A**, right) and platelet α-granules and dense granules **(B)**. Wild-type mice were treated with QLQX (0.468 g/kg/day, o.g.) or vehicle control for 7 days before analysis. *n* = 10. **(C-D)** QLQX administration (0.468 g/kg/day, 7 days, o.g.) in mice did not affect expression levels of platelet surface receptors, including αIIb (CD41), β3 (CD61), GPVI, and GPIbα (CD42b) as assessed by flow cytometry. Representative **(C)** and summary data **(D)** are shown, with values expressed as MFI. *n* = 5. **(E)** QLQX administration (0.468 g/kg/day, 7 days, o.g.) in mice inhibited platelet integrin αIIbβ3 activation (JON/A) and P-selectin exposure (CD62P) as detected by flow cytometry. *n* = 5. **(F)** Oral administration of QLQX to mice at doses of 0.234, 0.468, and 0.936 g/kg/day for 7 days concentration-dependently inhibited platelet aggregation induced by ADP (10, 20 µM), thrombin (0.03, 0.05 U/mL), and collagen (0.5, 1.0 µg/mL). *n* = 5. Statistical analyses were performed using unpaired two-tailed Student’s t-test in **(A-D)** and two-way ANOVA followed by Sidak’s multiple comparisons test in **(E-F)**. ns, not significant, **P < 0.01, ***P < 0.001

After QLQX was administered at a dosage of 0.468 g/kg (o.g.) per day for seven consecutive days, blood samples were collected from the mice to evaluate platelet function. The size and morphology of platelets, as well as the number of α-granules and dense granules, were comparable between QLQX-administered mice and vehicle-treated controls, as determined by transmission electron microscopy (TEM) (Fig. 3A-B). Flow cytometry analysis revealed that platelets from QLQX-treated mice presented normal expression levels of major platelet surface receptors, including αIIb (CD41), β3 (CD61), GPVI, and GPIbα (CD42b), compared with vehicle controls (Fig. 3C-D).

The effects of QLQX on platelet integrin αIIbβ3 activation and P-selectin exposure (a marker of α-granule release) were evaluated by flow cytometry, using JON/A (a specific monoclonal antibody targeting the activated conformation of αIIbβ3) and CD62P, respectively. Thrombin-induced αIIbβ3 activation and P-selectin exposure were reduced in platelets from mice receiving 7 days of QLQX (0.468 g/kg/day) treatment (Fig. 3E).

To further assess the antiplatelet efficacy of QLQX, three doses (0.234, 0.468, and 0.936 g/kg/day) within the previously established efficacy and safety range for rodents were administered [8, 10, 19]. QLQX dose-dependently inhibited platelet aggregation induced by ADP, thrombin, and collagen (Fig. 3F).

**Fig. 4:**
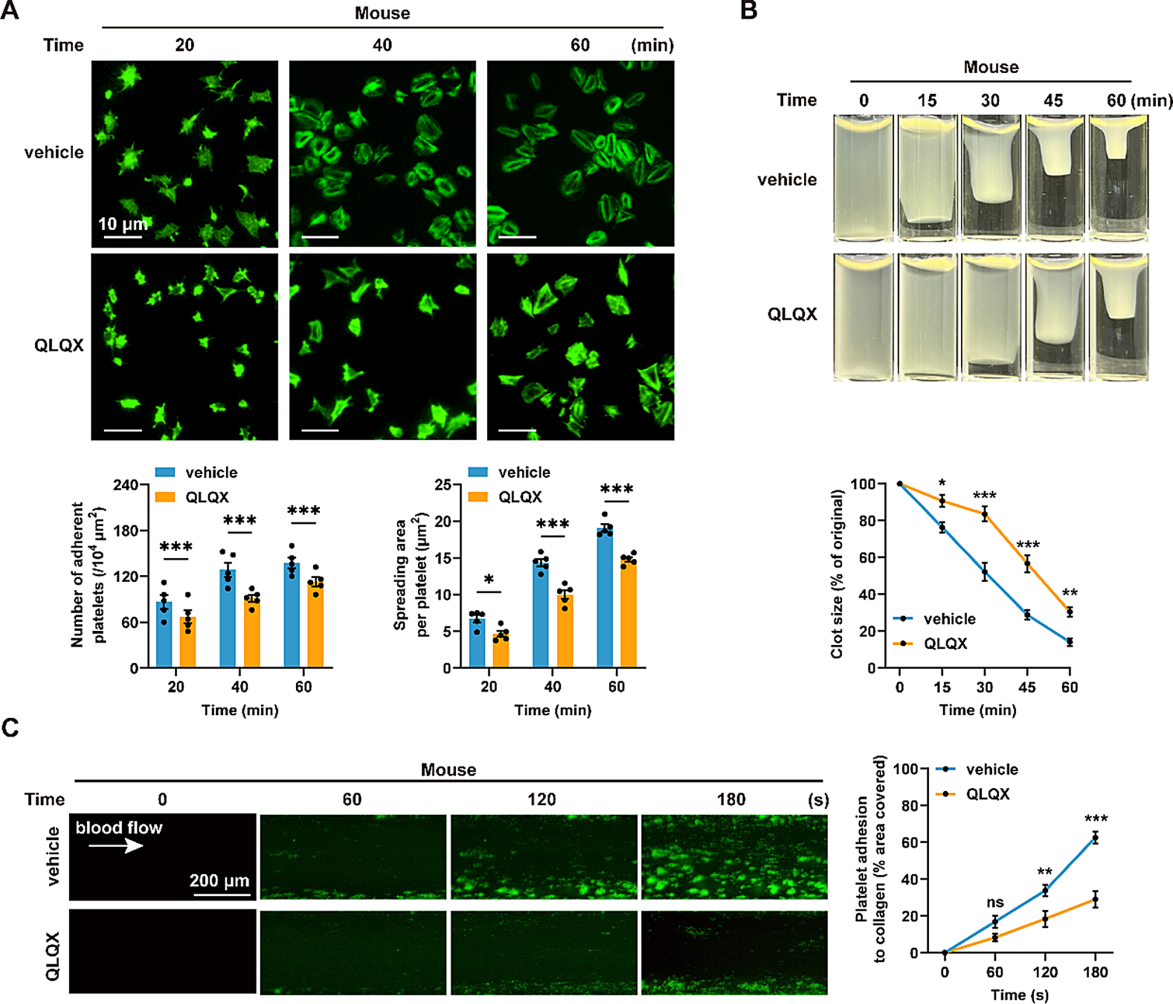
QLQX suppresses mouse platelet spreading, clot retraction, and ex vivo thrombus formation. **(A)** QLQX administration inhibited mouse platelet adherence and spreading on immobilized fibrinogen (100 µg/mL). Scale bar = 10 μm. **(B)** QLQX administration inhibited clot retraction induced by thrombin (1.0 U/mL) in mouse platelets. **(C)** QLQX administration inhibited ex vivo thrombus formation over an immobilized collagen surface at a shear rate of 1000 s^− 1^ in whole blood. Scale bar = 200 μm. Wild-type mice were randomly assigned to receive either QLQX (0.468 g/kg/day) or vehicle by oral gavage for 7 days. Statistical analyses were performed using two-way ANOVA followed by Sidak’s multiple comparisons test. *n* = 5. ns, not significant, *P < 0.05, **P < 0.01, ***P < 0.001

Further analysis revealed that platelets from QLQX-treated mice (0.468 g/kg/day for 7 days) exhibited reduced adhesion and spreading on immobilized fibrinogen compared with those from vehicle-treated controls (Fig. 4A). Consistently, QLQX treatment also significantly reduced thrombin-induced platelet clot retraction (Fig. 4B).

Furthermore, whole blood from QLQX‑ (0.468 g/kg/day) or vehicle‑treated mice perfused over collagen‑coated microchannels under arterial shear rate (1000 s⁻¹) demonstrated that QLQX significantly reduced thrombus formation area over the perfusion period (Fig. 4C).

In summary, QLQX exerts its antiplatelet effects by suppressing platelet activation without affecting platelet morphology, granule content, or key surface receptor expression.

### QLQX administration inhibits in vivo thrombus formation and alleviates microvascular obstruction in myocardial I/R injury

**Fig. 5:**
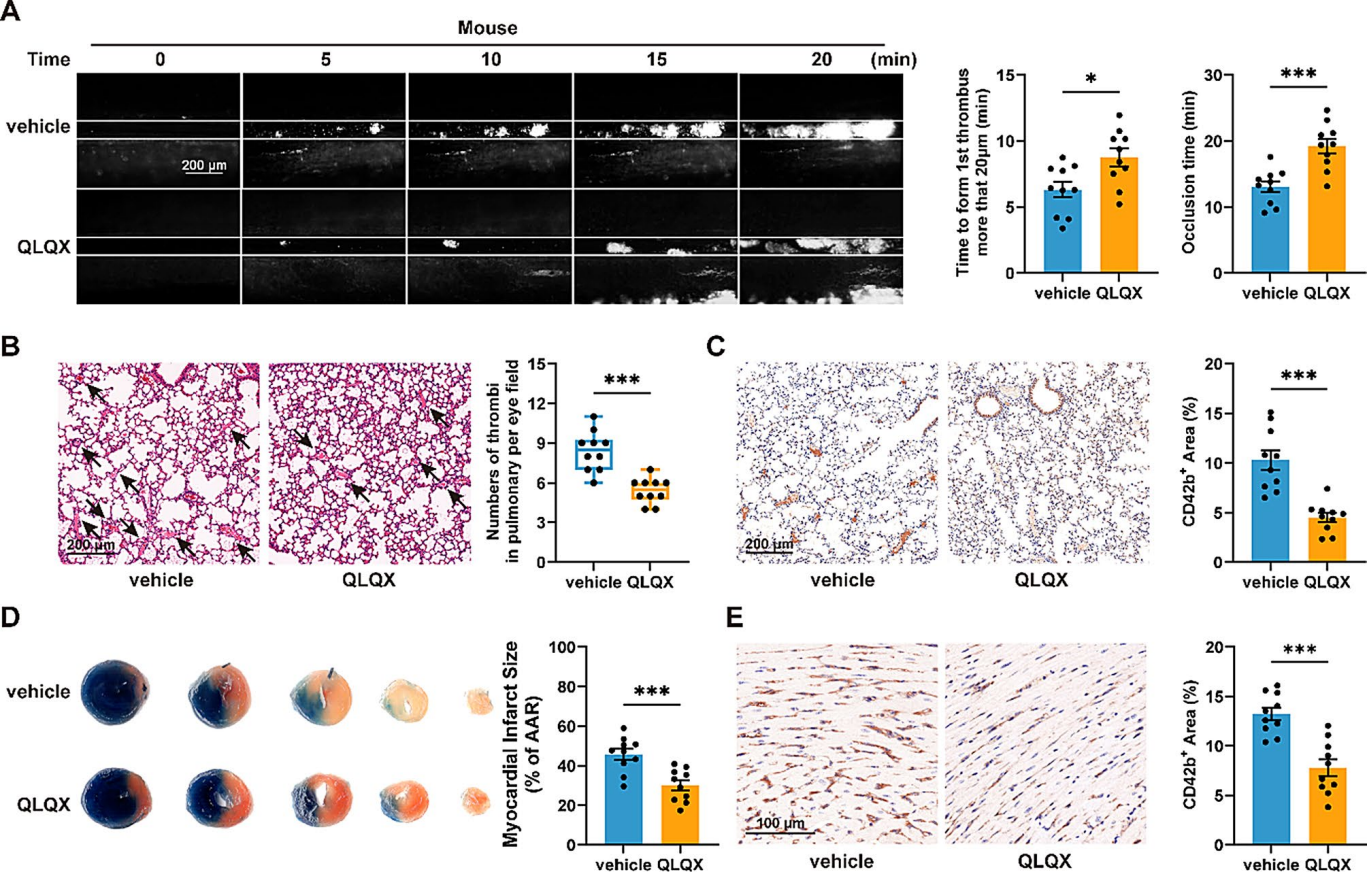
QLQX inhibits in vivo thrombus formation and microvascular obstruction in myocardial ischemia-reperfusion (I/R) injury. **(A)** QLQX administration inhibited FeCl_3_-induced thrombus formation in mice. Scale bar = 200 μm. **(B-C)** QLQX administration inhibited collagen and epinephrine-induced pulmonary embolism formation. H&E staining of lung sections, the arrows indicate emboli **(B)**, and immunohistochemistry staining of platelets using CD42b antibody in lung sections **(C)**. Scale bar = 200 μm. **(D-E)** QLQX administration decreased the infarct area/AAR ratio **(D)** and myocardial I/R injury-induced microthrombi (CD42b^+^) in the cardiac tissue **(E)** from mice after myocardial I/R injury at 48 h. Scale bar = 100 μm. Wild-type mice were randomly assigned to receive either QLQX (0.468 g/kg/day) or vehicle by oral gavage for 7 days. Data were analysed by unpaired two-tailed Student’s t test in **(A**,** C-E)** and Mann-Whitney U test in **(B)**. *n* = 10. ns, not significant, *P < 0.05, ***P < 0.001

To investigate the effect of QLQX on in vivo thrombosis, we employed FeCl_3_-induced mesenteric artery thrombosis and collagen/epinephrine-induced pulmonary embolism models in wild-type mice treated with QLQX (0.468 g/kg/day) via oral gavage for 7 days. In the FeCl_3_-induced thrombosis model, QLQX significantly attenuated thrombosis, as evidenced by prolonged time to first thrombus formation and stable occlusion (Fig. 5A). Consistently, in the pulmonary embolism model, QLQX reduced the number of pulmonary vascular thrombi (Fig. 5B) and decreased platelet accumulation in the lung, as indicated by smaller CD42b^+^ area (Fig. 5C).

Revascularization of occluded coronary arteries can precipitate myocardial I/R injury, in which platelets become hyperactivated and accumulate in the cardiac microcirculation [14, 20]. To further examine the impact of QLQX on microvascular thrombosis, we employed a myocardial I/R injury model in wild-type mice after treating them with QLQX (0.468 g/kg/day) for 7 days. Compared with vehicle treatment, QLQX reduced infarct size, as reflected by a lower infarct area/area-at-risk (AAR) ratio (Fig. 5D). Immunohistochemical staining of cardiac tissue sections with the platelet-specific marker CD42b revealed that QLQX treatment attenuated platelet accumulation induced by I/R (Fig. 5E).

### QLQX administration does not impair primary hemostasis or the coagulation

**Fig. 6:**
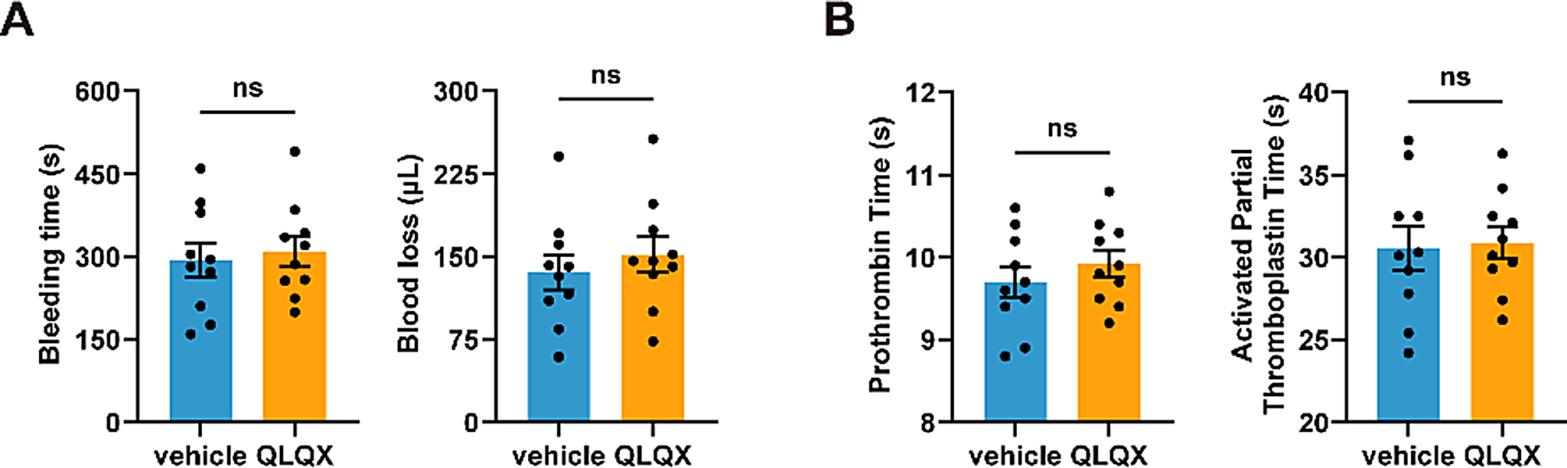
QLQX shows no significant effect on bleeding and coagulation parameters. **(A)** QLQX administration did not significantly alter bleeding time or blood loss. **(B)** QLQX administration did not significantly affect plasma prothrombin time (PT) or activated partial thromboplastin time (APTT). Wild-type mice were randomly assigned to receive either QLQX (0.468 g/kg/day) or the vehicle control by oral gavage for seven days. Data were analysed by an unpaired two-tailed Student’s t-test. *n* = 10. ns, not significant

To assess the impact of QLQX on physiological hemostasis, mice were administered the same regimen (0.468 g/kg/day for 7 days) used in the antithrombotic studies. The tail bleeding assay revealed that QLQX treatment did not significantly prolong bleeding time or increase blood loss compared with vehicle controls (Fig. 6A). Furthermore, QLQX treatment did not significantly increase either plasma prothrombin time (PT) or activated partial thromboplastin time (APTT) (Fig. 6B).

Collectively, these data indicate that QLQX suppresses thrombosis without compromising normal hemostasis or inducing coagulopathy.

### QLQX administration suppresses platelet Ca^2+^ influx and PKC signaling pathway

**Fig. 7:**
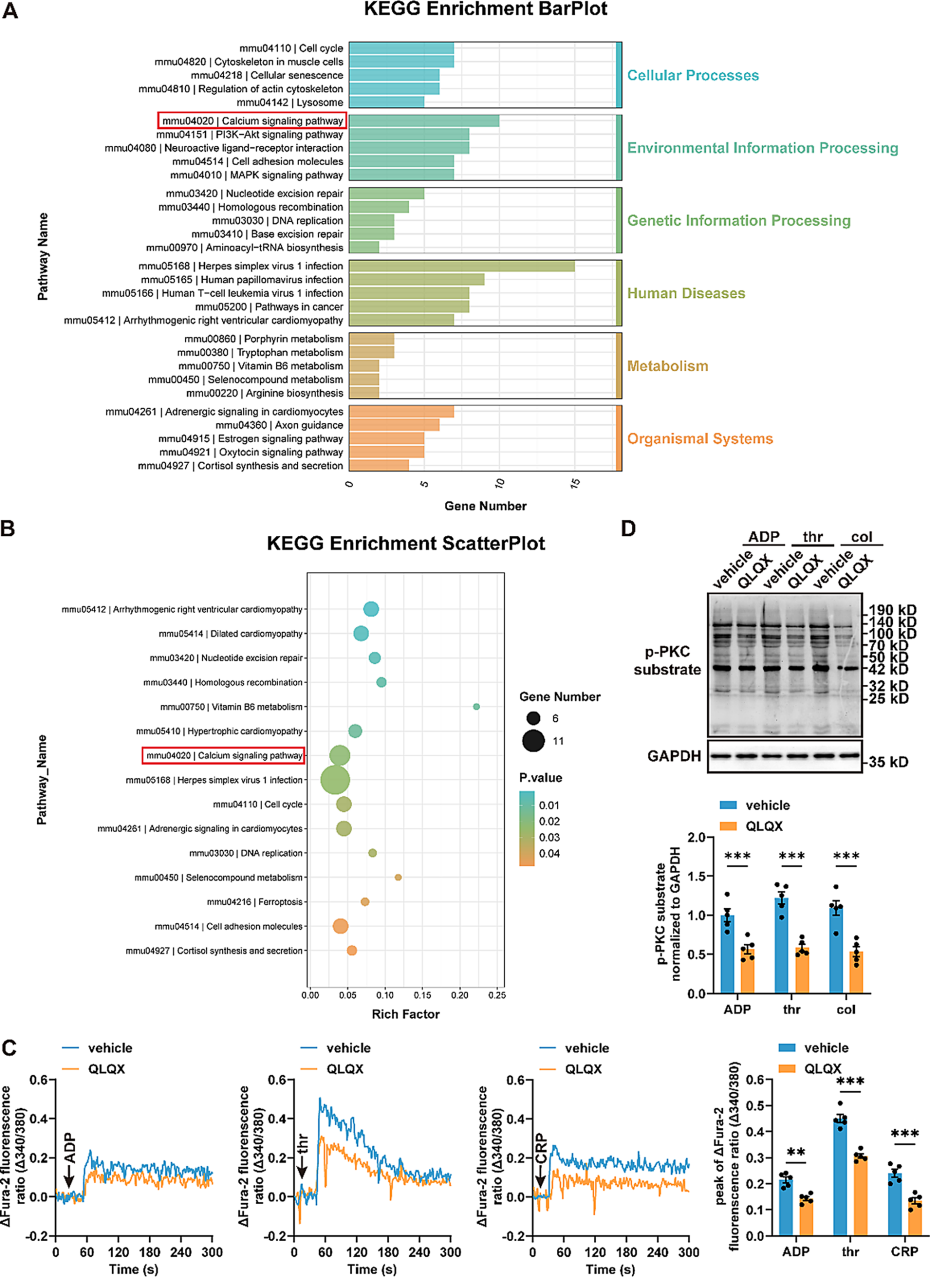
QLQX suppresses platelet Ca^2+^ influx and PKC phosphorylation **(A-B)** Platelet RNA sequencing (RNA-seq) was performed on platelets of QLQX- or vehicle-treated mice. n = 3. KEGG enrichment pathway annotation results are shown, with the six first-level KEGG pathways distinguished in different colors **(A)**. Bubble plot of KEGG enrichment analysis shows the top 15 KEGG pathway enrichment results for differentially expressed genes **(B)**. **(C)** QLQX administration inhibited Ca^2+^ influx in washed platelets stimulated with 10 μM ADP, 0.05 U/mL thrombin, or 0.5 μg/mL collagen-related peptide (CRP). n = 5. **(D)** Western blot analysis shows that QLQX inhibits PKC substrates phosphorylation in washed platelets stimulated with 10 μM ADP, 0.05 U/mL thrombin, or 0.5 μg/mL collagen. n = 5. Wild-type mice were randomly assigned to receive either QLQX (0.468 g/kg/day) or the vehicle control by oral gavage for seven days. Statistical analyses were performed using two-way ANOVA followed by Sidak’s multiple comparisons test in **(C-D)**. **P < 0.01, ***P < 0.001

In order to investigate the mechanism by which QLQX inhibits platelet activation, RNA sequencing was conducted on platelets obtained from wild-type mice that had been administered QLQX (0.468 g/kg/day for seven days) or the vehicle control. The results showed that 216 genes were upregulated and 316 genes were downregulated. Kyoto Encyclopedia of Genes and Genomes (KEGG) pathway enrichment analysis revealed a significant overrepresentation of differentially expressed genes (DEGs) associated with the calcium signaling pathway (Fig. 7A-B). Ca^2+^-dependent cellular signaling is a pivotal regulator throughout platelet activation, controlled primarily by store-operated Ca^2+^ influx and PLC-PKC signaling pathway [21]. Accordingly, we monitored platelet Ca^2+^ mobilization in real time following agonist stimulation using spectrofluorometry. The results showed that QLQX administration inhibited Ca^2+^ influx in mouse platelets stimulated with ADP, thrombin, and collagen-related peptide (CRP) (Fig. 7C). In parallel, Western blotting analysis showed that QLQX suppressed PKC phosphorylation in platelets under stimulation with ADP, thrombin, and collagen (Fig. 7D).

Therefore, QLQX exerts its inhibitory effect on platelet activation by suppressing the Ca²⁺ and PKC signaling triggered by agonists.

## Discussion

Heart failure is a recognized prothrombotic state, which confers elevated risks of thromboembolism, stroke, and death on patients with HF [22]. QLQX has demonstrated clinical benefit in heart failure; however, its effects on platelet activation and thrombosis remain unclear. In this study, we demonstrated that: (1) QLQX reduces platelet aggregation, spreading, clot retraction, and ex vivo thrombus formation in patients with CHF; (2) QLQX inhibits platelet activation in wild-type mice; (3) QLQX attenuates FeCl_3_-induced mesenteric arteriole thrombosis and collagen/epinephrine-induced pulmonary embolism, and reduces microvascular thrombosis and infarct size after myocardial I/R, without increasing bleeding in mice; (4) mechanistically, QLQX inhibit platelet Ca²⁺ influx and PKC signaling pathway.

Abundant evidence indicates heightened platelet activation in HF, characterized by increased whole‑blood aggregability and upregulated platelet activation molecules (e.g., GPIIb/IIIa, P-selectin) [22, 23]. Given that HF frequently coexists with coronary heart disease (CHD), and platelet hyperreactivity may further elevate thrombotic risk, leading to accelerated disease progression and worsened clinical outcomes [24]. However, current antiplatelet therapy, including widely used aspirin and clopidogrel, has demonstrated efficacy in many cardiac diseases, whereas the risk of major bleeding outweighs the antithrombotic benefit [25]. However, the role of antiplatelet treatments in reducing thromboembolic complications in CHF remains debated, and there is insufficient evidence to support routine use of antiplatelet agents [22]. Our data demonstrate that QLQX administration reduces platelet activation in patients with CHF and exerts antithrombotic effects without increasing bleeding in mice, suggesting a potential therapeutic strategy for managing thrombosis in patients with CHF.

As a multi-herb TCM formulated based on the classical compatibility theory “Monarch-Minister-Assistant-Guide”, QLQX is composed of *Astragali Radix*,* Ginseng Radix et Rhizoma*,* Aconiti Lateralis Radix Praeparata*,* Salviae Miltiorrhizae Radix et Rhizoma*,* Descurainiea Semen*,* Alismatis Rhizoma*,* Polygonati Odorati Rhizoma*,* Cinnamomi Ramulus*,* Carthami Flos*,* Citri Reticulatae Pericarpium*,* and Periplocae Cortex* [8]. Previous studies have identified astragaloside IV (from the monarch herb *Astragali Radix*) and ginsenoside Rb1 (from the minister herb *Ginseng Radix et Rhizoma*) as two principal bioactive components of QLQX—both compounds were detected at high concentrations in plasma and cardiac tissue following oral administration [26, 27]. Ginsenoside Rb1 and its derivative Rg5 have been shown to effectively inhibit Ca²⁺-dependent platelet activation, granule secretion, and arterial thrombosis [28, 29]. Meanwhile, *Astragali Radix* has demonstrated antithrombotic effects in both deep vein thrombosis and tail thrombosis models [30, 31], and its principal component, Astragaloside IV, targets signaling pathways such as PI3K/Akt and Ca²⁺ influx [32, 33], which are central to thrombus formation. Consistently, our results showed that QLQX exerted a favorable antithrombotic effect by suppressing Ca²⁺ influx and PKC activation. Therefore, astragaloside IV and ginsenoside Rb1 may be bioactive components that underlie the antiplatelet activity of QLQX. Notably, this does not exclude the potential contributions of other components (e.g., salvianolic acid B from *Salviae Miltiorrhizae Radix et Rhizoma*, which also exhibits mild antiplatelet effects) [34], as the holistic efficacy of TCM formulations relies on the synergism of multiple components.

Our study showed that QLQX effectively inhibited platelet activation without increasing bleeding time and blood loss. This favorable dissociation is supported by preserved blood counts, surface expression of key platelet receptors (GPIbα, GPVI, and αIIbβ3), and coagulation parameters (PT/APTT). A plausible explanation lies in its multi-component nature. While some bioactive compounds inhibit platelet function, others may counteract the tendency to bleed during vascular injury. Notably, ginsenoside Rb1, a principal bioactive component of QLQX, has been reported to inhibit platelet function without prolonging bleeding time [28]. This further supports the concept that QLQX attenuates excessive, agonist-driven platelet activation relevant to thrombosis, while preserving basal platelet function to support normal haemostasis. Future studies need to explore the combined regulatory mechanisms of these components on platelet function.

QLQX has demonstrated substantial clinical efficacy in the management of HF [6, 12]. A landmark trial involving 3119 patients with HF reported that QLQX reduced the composite endpoint of cardiovascular death and HF hospitalization by 22% [12]. Extensive preclinical studies further substantiate its cardioprotective benefits across models of acute and chronic injury, including the attenuation of myocardial and endothelial dysfunction and the promotion of microangiogenesis [35–37]. The novel antiplatelet properties of QLQX identified in our study extend this mechanistic understanding. By not only alleviating cardiac injury but also mitigating the concomitant thrombotic risk common in HF, QLQX’s multifaceted action offers a coherent mechanistic basis for its efficacy in reducing major adverse cardiovascular events.

Prior studies have shown QLQX exerts cardioprotective effects through multiple molecular mechanisms: it eases oxidative stress in cardiomyocytes through PI3K/Akt/mTOR/HIF-1α/NRF2 signaling [37, 38], alleviates cardiomyocyte apoptosis through SIRT1/AMPK/PGC‑1α signaling [37], and adjusts calcium transients and sparks in cardiomyocytes to slow beating rate [39]. Our study extends QLQX’s regulatory role to platelets, demonstrating that QLQX inhibits platelet activation by suppressing agonist-induced Ca²⁺ influx and PKC activation. In platelets, Ca^2+^ influx is critical for key activation processes. Upon agonist stimulation, platelets release Ca²⁺ from the dense tubular system—a process involving the activation of PKC, thereby triggering αIIbβ3 activation [21]. Dysregulated platelet Ca^2+^ influx has been documented in frail elderly adults and in patients with atrial fibrillation, diabetes, and ischemic heart disease, which increases thrombotic risk [40, 41]. These observations highlight platelet Ca^2+^ pathways as attractive therapeutic targets.

## Conclusion

Our data indicated that QLQX attenuates platelet activation and thrombus formation. The antiplatelet activity of QLQX is primarily mediated by suppressing Ca²⁺ influx and PKC activation. These findings suggest that QLQX represents a potential therapeutic strategy for managing thrombotic complications. Further studies are needed to explore the antiplatelet effect of QLQX in diverse models. Additionally, advanced component analysis techniques may further clarify the bioactive constituents and mechanisms of QLQX.

## Supplementary Information

Below is the link to the electronic supplementary material.

Supplementary Material 1

Supplementary Material 2

## Data Availability

The full RNA sequencing data are available at Sequence Read Archive (SRA) with accession number PRJNA1372821.

## Abbreviations

AAR: Area at risk
CHD: Coronary heart disease
CHF: Chronic heart failure
DEGs: Differentially expressed genes
HF: Heart failure
I/R: Ischemia-reperfusion
KEGG: Kyoto Encyclopedia of Genes and Genomes
LAD: Left anterior descending
MACE: Major adverse cardiovascular events
PRP: Platelet‑rich plasma
PKC: Protein kinase C
QLQX: Qiliqiangxin capsules
TCM: Traditional Chinese medicine
