# Supplementary material for: Qiliqiangxin capsule attenuates platelet activation and thrombosis by suppressing Ca2+ influx and PKC signaling
Source: Thromb J. 2026 Jan 2;24:2. doi: 10.1186/s12959-025-00823-8 (PMC12772080; doi:10.1186/s12959-025-00823-8)
Supplement: Supplementary file 1 — Supplementary Material 1 [file 12959_2025_823_MOESM1_ESM.docx]

**Supplementary data**

**Table S1. Reagents and antibodies**

| REAGENTS | SOURCES | IDENTIFIER |
| --- | --- | --- |
| Antibodies |  |  |
| phospho-PKC substrate antibody (1:1000) | Cell Signaling Technology | 2261 |
| GAPDH Rabbit mAb (1:5000) | Cell Signaling Technology | 2118 |
| HRP-conjugated Goat Anti-Rabbit IgG (H+L) (1:5000) | Proteintech | RGAR001 |
| Rabbit Recombinant Monoclonal CD42b antibody (1:200) | Abcam | ab183345 |
| Goat Anti-Rabbit IgG H&L (Alexa Fluor® 555) | Abcam | ab150078 |
| APC-conjugated CD41 Antibody | BioLegend | 133914 |
| PE-conjugated CD61 Antibody | Invitrogen | MA5-16917 |
| PE-conjugated GPVI Antibody | R&D Systems | FAB6758P |
| PE-conjugated CD62PAntibody | BioLegend | 148305 |
| PE-conjugated JON/A | emfret | M023-2 |
| Chemicals | | |
| QLQX capsules | Shijiazhuang Yiling Pharmaceutical Co. | / |
| ADP | Chrono-Log | P/N384 |
| Collagen | Chrono-Log | P/N385 |
| Thrombin | Chrono-Log | P/N386 |
| epinephrine | Chrono-Log | P/N393 |
| Apyrase | Sigma Aldrich | A6535 |
| Fibrinogen | Sigma Aldrich | F3879 |
| Calcein-AM | Sigma Aldrich | 206700 |
| FeCl_3_ | Sigma-Aldrich | 157740 |
| Evans blue dye | Sigma-Aldrich | E2129 |
| TTC | Sigma-Aldrich | 17779 |
| TRIzol Reagent | Sigma-Aldrich | T9424 |
| Hematoxylin | Sigma Aldrich | MHS32 |
| Eosin | Sigma Aldrich | 318906 |
| Fura-2 AM | Thermo Fisher Scientific | F1221 |
| Phalloidin-488 | Thermo Fisher Scientific | A12379 |

**Table S2. Baseline characteristics of the study population.**

| **Characteristics** | **CHF patients (n = 10)** |
| --- | --- |
| Age,years | 57.80 ± 10.10 |
| Male | 7 (70.00%) |
| BMI | 26.33 ± 5.33 |
| History of smoking | 0 (0.00%) |
| History of drinking | 0 (0.00%) |
| **Medical history (n (%))** |  |
| Hypertension | 6 (60.00%) |
| Diabetes mellitus | 2 (2.00%) |
| Atrial fibrillation | 0 (0.00%) |
| Myocardial infarction | 0 (0.00%) |
| Stroke | 0 (0.00%) |
| Fatty liver | 0 (0.00%) |
| **Platelet parameters** |  |
| Platalet count, ×10^9^ | 252.10 ± 31.76 |
| MPV, fL | 8.91 ± 0.85 |
| PCT, % | 0.23 (0.18-0.27) |
| PDW, % | 16.68 (16.10-17.20) |

Values are presented as mean ± SD, percentage (%), or median (interquartile range). Statistical analyses were performed using Student’s t-test or Mann-Whitney U test. BMI: Body Mass Index, MPV: Mean Platelet Volume, PCT: Plateletcrit, PDW: Platelet Distribution Width.
