# Supplementary figures and images for: Qiliqiangxin capsule attenuates platelet activation and thrombosis by suppressing Ca2+ influx and PKC signaling
Source: Thromb J. 2026 Jan 2;24:2. doi: 10.1186/s12959-025-00823-8 (PMC12772080; doi:10.1186/s12959-025-00823-8)

## Original, unedited images for Figure 4

p-PKC substrate for Fig.4D

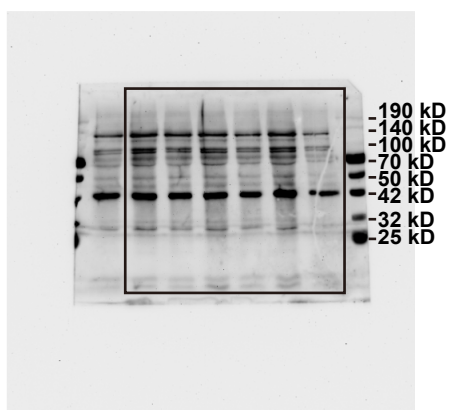

GAPDH for Fig.4D

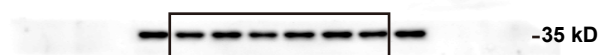

Supplement: Supplementary file 2 — Supplementary Material 2 [file 12959_2025_823_MOESM2_ESM.pdf]
